# Supplementary material for: Haplotype Tagging Sheds Light on Speciation Between Two Divergent Cryptic Species of the Brown Algae Ectocarpus
Source: Genome Biol Evol. 2026 Oct 22;18(11):evag268. doi: 10.1093/gbe/evag268 (PMC13756086; doi:10.1093/gbe/evag268)
Supplement: Supplementary file 1 [file evag268_supplementary_data.zip › Supl_Figures_R1.pdf]

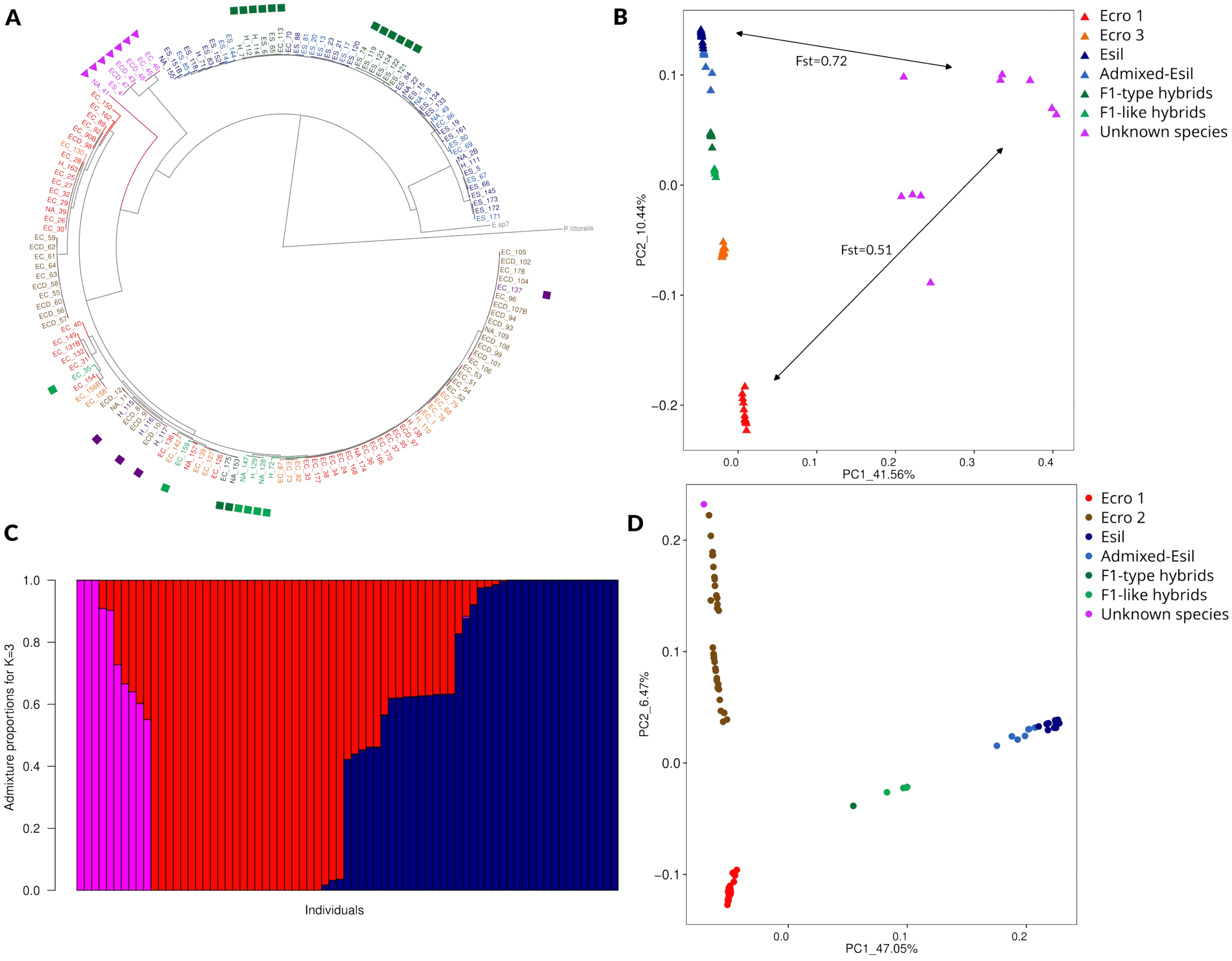

Figure S1. A) Neighbor-Joining tree of mitochondrial genomes. The tree depicts the relationships among 154 out of the 160 individuals from this study, with *Ectocarpus* sp7 (the reference genome species) and *Pilayella littoralis* as outgroups. Individual names are coloured according to genomic cluster: *E. siliculosus* (shades of blue), *E. crouaniorum* (shades of red), putative undescribed species (magenta, marked by triangles), Esil × Ecro hybrids (green, marked by squares), and hybrids between the unknown species and *E. crouaniorum* (purple, marked by squares). B) Principal component analysis (PCA), showing cryptic lineage differentiation within the *Ectocarpus* complex in Europe (n=73). Individuals are coloured according to the NGSadmix and global PCA results; *E. siliculosus* in blue, *E. crouaniorum* in red, putative hybrids in green and the unknown *Ectocarpus* species in magenta. Genetic structure based on NGSadmix analysis (K=3, n=73) in Europe showing the two parental species (*E. crouaniorum* in red and *E. siliculosus* in blue) and the unknown *Ectocarpus* species in magenta. C) Genetic structure based on NGSadmix analysis (K=3, n=73) in Europe showing the two parental species (*E. crouaniorum* in red and *E. siliculosus* in blue) and the unknown *Ectocarpus* species in magenta. D) Principal component analysis (PCA), showing cryptic lineage differentiation within the *Ectocarpus* complex in Chile (n=87).
Alt-text: Mitochondrial phylogeny, PCA, and genomic ancestry reveal unknown *Ectocarpus* lineage in Europe and Chile.

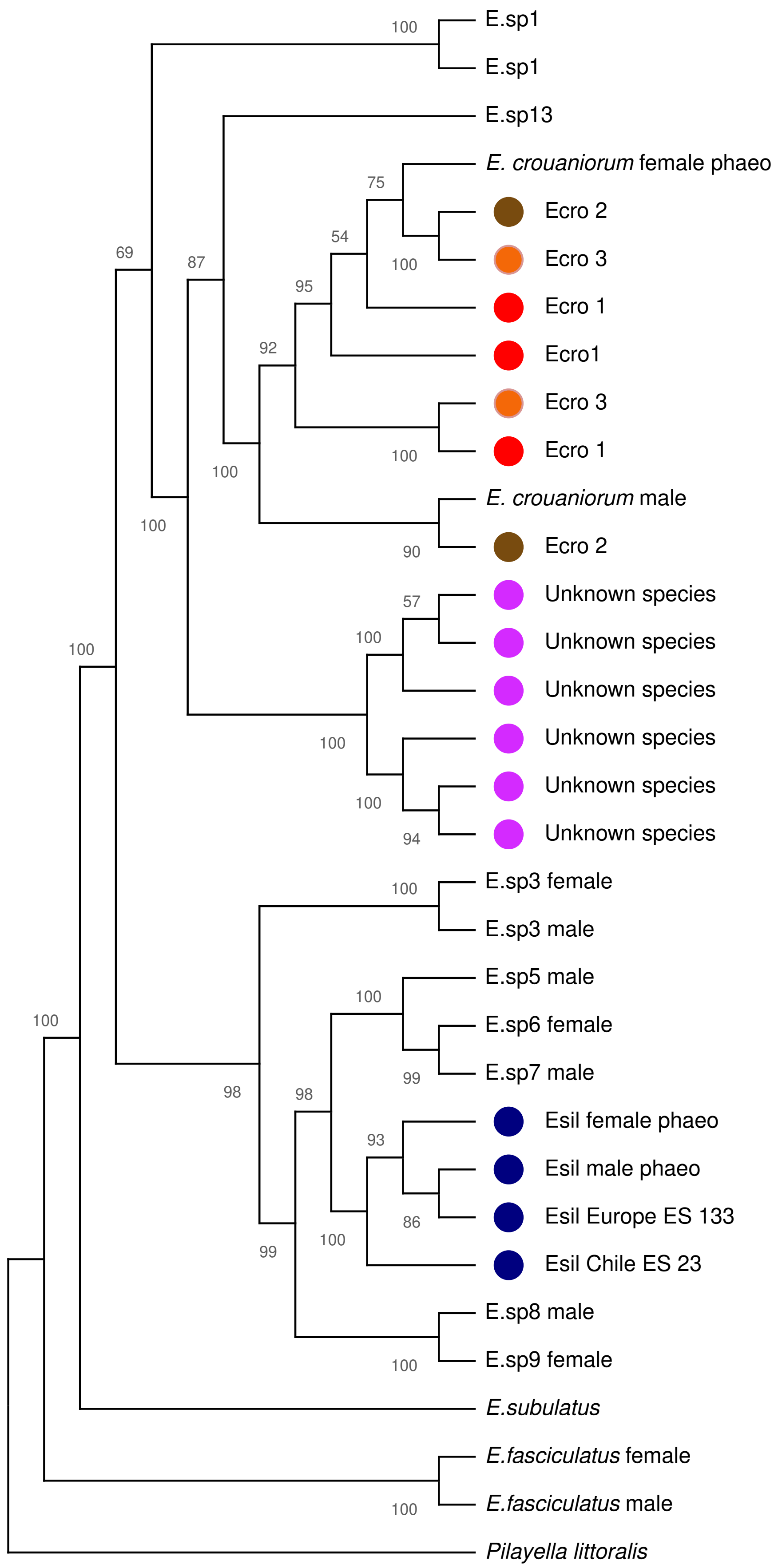

Figure S2. Neighbor-Joining tree of mitochondrial genomes. The tree depicts the relationships among some representative individuals from this study (marked in colored circles), with 12 recognized species of *Ectocarpus* (Denoeud et al., 2024) including *Ectocarpus* sp7 (the reference genome species) and *Pilayella littoralis* as outgroups. Circles are coloured according to genomic clusters: *E. siliculosus* (blue), *E. crouaniorum* (shades of red), putative undescribed species (magenta).
Alt-text: Position of focal lineages within the *Ectocarpus* mitochondrial phylogeny.

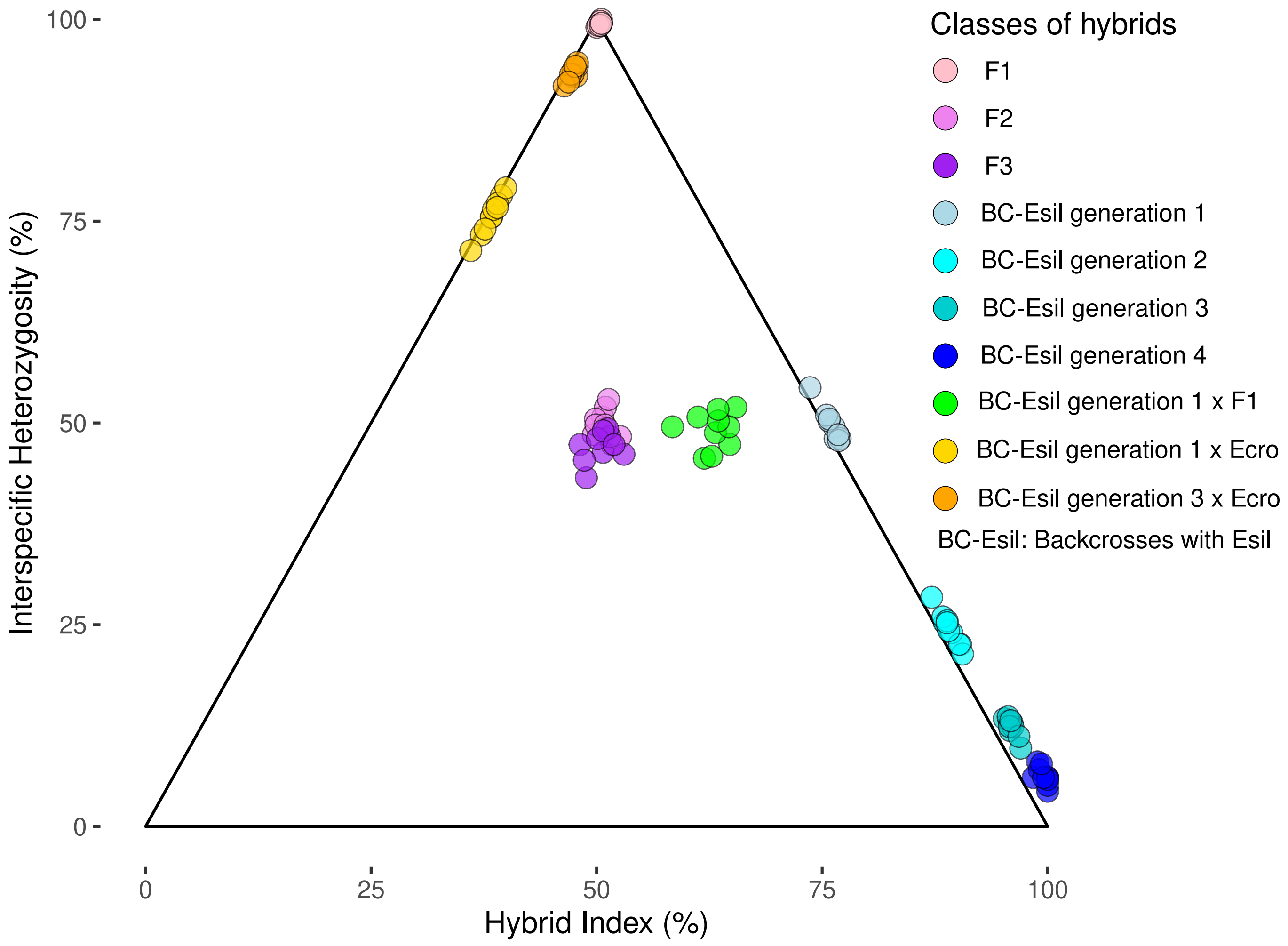

Figure S3. Simulated individuals were derived from pure Ecro1–Esil and Ecro3–Esil lineages in Chile and Europe, respectively and 2,291 species-specific SNPs. Ten individuals were generated per hybrid class and visualized in a triangle plot. The axis of the hybrid index is relative to the *E. siliculosus* species. Only one direction of backcrossing is shown for clarity, as the reciprocal is symmetric.

Alt-text: Triangle plots of simulated hybrid and backcross classes based on hybrid index and interspecific heterozygosity.

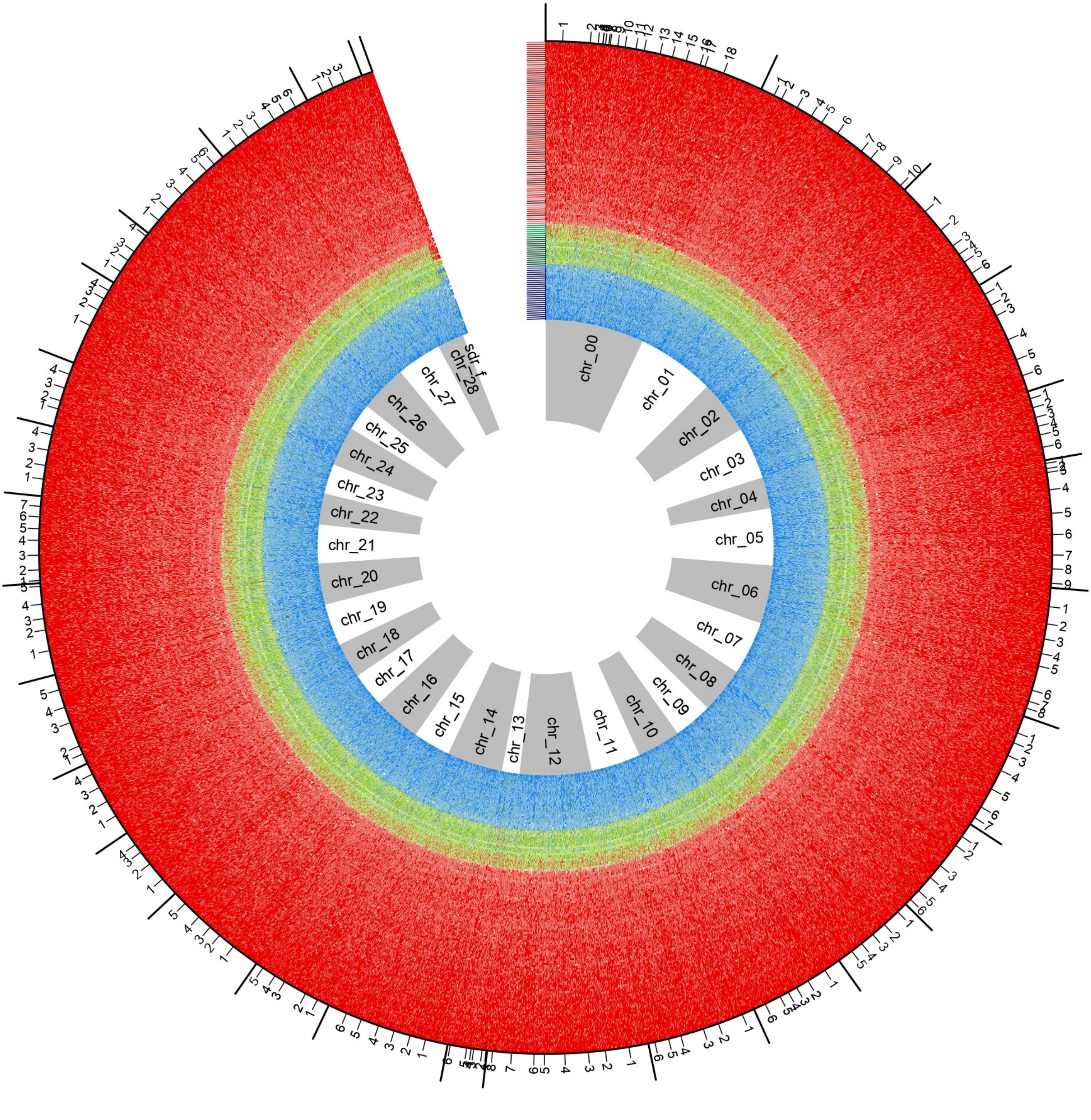

Figure S4. Circular representation of the location of barriers and the polarity of diagnostic markers for all samples (n=137) across chromosomes. The inner ring shows the location of each chromosome in alternating grey and white. Moving outwards, the next ring indicates Esil parental lineage (in blue), then the admixed Esil (light blue), the F1-type hybrids (dark green), the F1-like (light green), and Ecro parental lineage (in red). Each ring shows the genotypes of each sample at each diagnostic marker (p>0.6). Red bars are diagnostic of Ecro, blue bars are diagnostic of Esil, and green bars are heterozygous for markers diagnostic of each species. The location of each 50 kb of sequence for each chromosome is indicated on the outside of the circle.

Alt-text: Chromosome-wide diagnostic marker profiles across parental, admixed, and hybrid individuals.

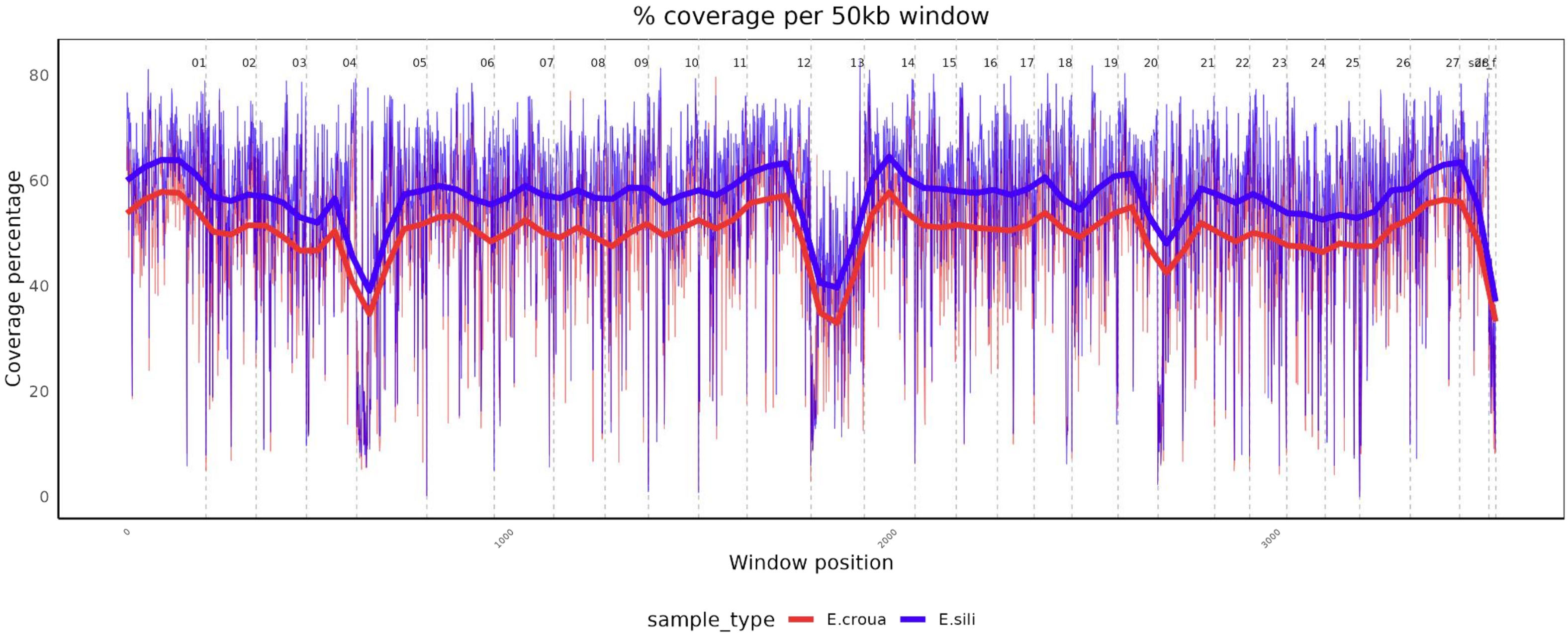

Figure S5. Mean mapping coverage depth for each pure species (Ecro N=81, Esil N=26) calculated in 50kb windows across the reference genome assembly of *Ectocarpus* sp.7. Thin colored lines represent the observed mean coverage values per window, while thicker smoothed curves (LOESS smoothing, span = 0.07) highlight large-scale coverage trends along the genome. Dashed vertical lines indicate chromosome boundaries. *E. crouaniorum* in red, *E. siliculosus* in blue.

Alt-text: Genome-wide mapping coverage depth across *E. crouaniorum* and *E. siliculosus*.

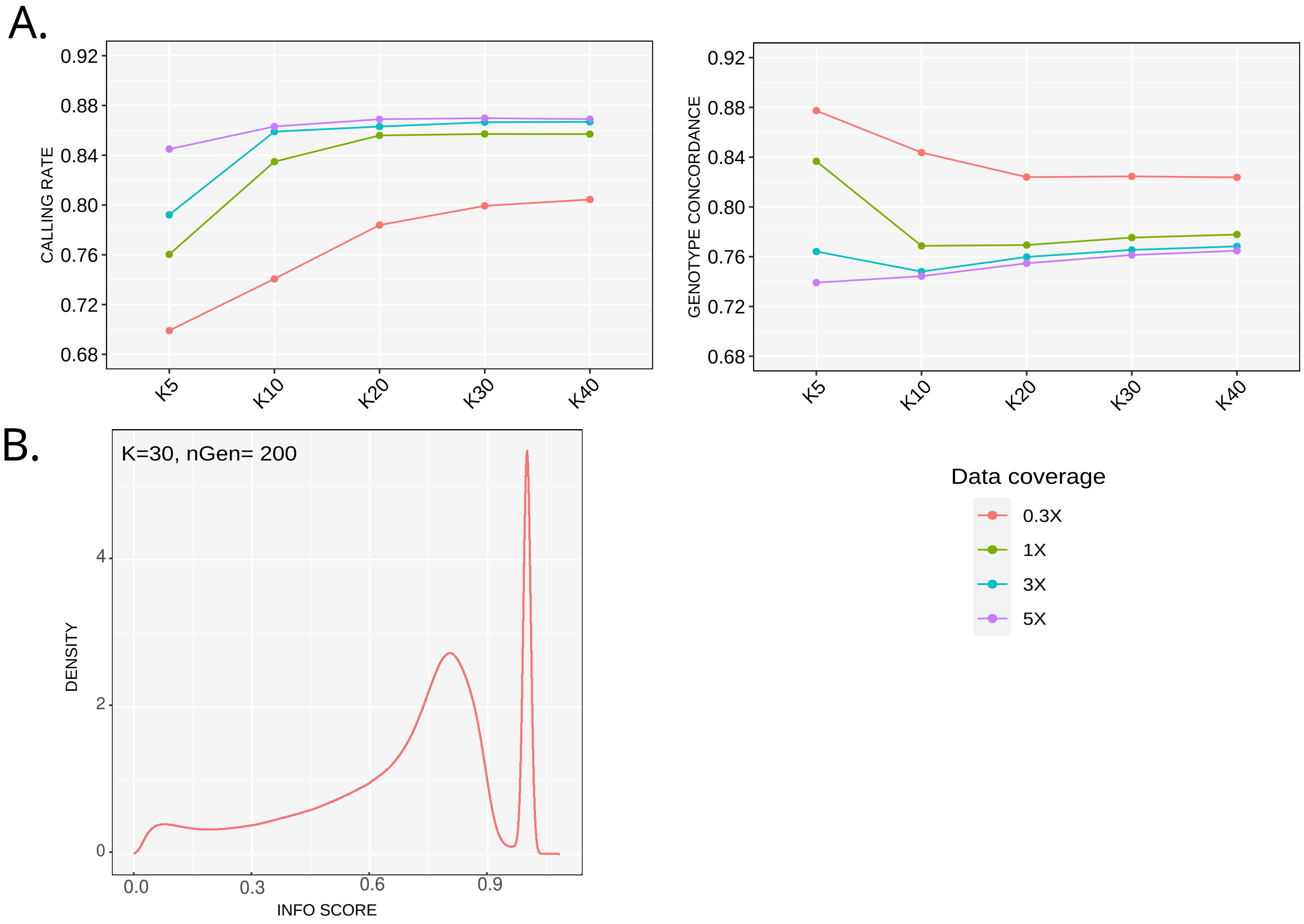

Figure S6. Choice of parameters for genotype imputation using STITCH. A) The parameter K, representing the number of ancestral haplotypes, was optimized by testing eight individuals with higher sequencing depth (5.8-8.3x) representing both species and potential hybrids. These individuals were down-sampled with Picard to reach an average depth of 0.3X, 1X, 3X, and 5X to mimic the lowest coverages in the dataset. Each dot corresponds to the average of all individuals and is colored according to the depth. Genotype imputation was then performed with STITCH on a subset of chromosomes (1-4), testing different values of K (5, 10, 20, 30, and 40). Performance was assessed by comparing genotype concordance (imputed genotype being equal to the hidden genotype) and calling rate (percentage of retrieved genotypes). Note that genotype concordance includes non-called genotypes (./.) and thus needs to be considered alongside the calling rate. These metrics were calculated using SNPs filtered with IS > 0.4, AD > 5, and quality ≥ 40. B) Distribution of the imputation info score (IS) used to define a filtering threshold. Shown here is an example for K = 30 and ngen = 200 across the four subset of chromosomes used for parameter optimization.
Alt-text: Optimization of STITCH parameters for genotype imputation using our data.
